# Increased risk of stroke among patients with inflammatory bowel disease: A PRISMA‐compliant meta‐analysis

**DOI:** 10.1002/brb3.2159

**Published:** 2021-05-07

**Authors:** Yao Chen, Xiang Wang

**Affiliations:** ^1^ Department of Internal Medicine Hospital of Nanjing University of Science and Technology Nanjing China; ^2^ Department of Ultrasound The Third Affiliated Hospital of Chongqing Medical University (Gener Hospital) Chongqing China

**Keywords:** inflammatory bowel disease, meta‐analysis, stroke

## Abstract

**Background:**

Previous studies on the association between inflammatory bowel disease (IBD) and stroke showed conflicting results.

**Methods:**

Articles published before July 2020 were searched in databases (PubMed, Web of Science, Medline, EMBASE, and Google Scholar). We computed all multivariate odds ratios (ORs) or relative risks (RRs) and 95% confidence intervals (CI) by using STATA 12.0 software.

**Results:**

The meta‐analysis indicated that IBD was associated with an elevated risk of stroke (OR/RR = 1.21, 95% CI 1.08 to 1.34, *I* ^2^ = 83.6%, *p* < .001). In addition, both Crohn's disease (CD) and ulcerative colitis (UC) were associated with a higher risk of stroke (CD: OR/RR = 1.25, 95% CI 1.03 to 1.52, *I* ^2^ = 86.1%, *p* < .001; UC: OR/RR = 1.09, 95% CI 1.04 to 1.15, *I* ^2^ = 54.7%, *p* = .051). Subgroup study showed that IBD was associated with a higher risk of stroke in cohort studies (RR = 1.21, 95% CI 1.08 to 1.36, *I* ^2^ = 85.0%, *p* < .001). Subgroup study showed that IBD was related to an elevated risk of stroke in both Caucasian and Asian groups (Caucasian group: OR/RR = 1.13, 95% CI 1.05 to 1.23, *I* ^2^ = 44.6%, *p* = .094; Asian group: OR/RR =1.36, 95% CI 1.07 to 1.74, *I* ^2^ = 92.5%, *p* < .001).

**Conclusion:**

IBD is a risk factor for stroke. More high‐quality large‐sample epidemiologic studies about the relationship between IBD and stroke should be further conducted.

## Graphical abstract

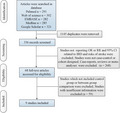

## 1 INTRODUCTION

Inflammatory bowel disease (IBD) is a chronic inflammatory disorder of the intestine, which includes two varieties, Crohn's disease (CD) and ulcerative colitis (UC) (Greuter & Vavricka, 2019). It has been reported that the incidence and prevalence of IBD are increasing around the world with a prevalence rate of 8.3 per 1,000 of the population in North America (Sairenji et al., 2017). The main clinical symptoms are abdominal pain and diarrhea (Wehkamp et al., 2016). The etiology of IBD remains unclear. Epidemiological data suggested that multiple risk factors are associated with IBD, such as diet, smoking, and genetic factor (Malik, 2015).

Stroke is a cerebrovascular disorder with high incidence rate, high disability rate and high mortality, and characterized by the sudden onset of symptoms and clinical signs (Boursin et al., 2018). Stroke is the leading cause of disability as well as the second highest cause of death worldwide (Campbell et al., 2019). Ischemic stroke caused by arterial occlusion accounts for 87% of all stroke cases (Koh & Park, 2017). Data indicated 2.4 million new stroke cases and 1.1 million deaths caused by stroke per year in China, 1.1 million cases of stroke in Europe (Béjot et al., 2016; Wu et al., 2019). Although the incidence of new and recurrent stroke is declining globally due to the use of specific prevention medications, the burden of stroke is still increasing because of population aging and the increasing incidence rate of stroke in young adults (Guzik & Bushnell, 2017; Hankey, 2017).

Previous studies showed an increased risk of stroke in patients with psoriasis, systemic sclerosis, and systemic lupus erythematosus (Gu et al., 2019; Raaby et al., 2017; Ungprasert et al., 2016). Besides, studies showed that the elevated concentrations of inflammatory factors such as C‐reactive protein were associated with higher risk of stroke, which suggested that inflammatory mechanisms may play a pivotal role in the progression of stroke (Kaptoge et al., 2010; Zhang et al., 2019). However, previous studies on the association between IBD and stroke showed conflicting results (Dregan et al., 2014; Huang et al., 2014). Therefore, we conducted this systemic review and analyzed the collected data to find out whether IBD was the risk factor of stroke or not.

## 2 METHODS

The study was performed on the basis of the Preferred Reporting Items for Systematic reviews and Meta‐Analysis (PRISMA) guideline (Moher et al., 2009).

### 2.1 Search strategy and selection criteria

We used search terms as follows: (“inflammatory bowel disease” OR “ulcerative colitis” OR “Crohn's disease”) AND (“stroke”). Articles published before July 2020 were searched in the following databases: PubMed, Web of Science, Medline, EMBASE, and Google Scholar. After eliminating duplicates, 336 studies were included in the study. Selection criteria were showed as follows: (1) Included studies should report odds ratio (OR) in case–control studies or relative risk (RR) in cohort studies and 95% confidence intervals (CI) related to IBD and risks of stroke. (2) We also included studies when the RR or OR and 95%CI could be calculated from the data in the studies. In addition, studies should not be case reports, reviews, or meta‐analyses.

### 2.2 Data extraction

Two investigators read the titles, abstracts, and full texts of included studies independently. We included data as follows: author, publication year, study design, study location, sample sizes, event for analysis, adjustment factors, and results.

### 2.3 Meta‐analysis

We computed all the results in included studies by using STATA 12.0 software. We used Q test and inconsistency index (*I* ^2^) to evaluate heterogeneities between studies. When the heterogeneity was low (*p* value for Q test >0.05 and *I* ^2^ < 50%), fixed effects models were used to compute all the results; when the heterogeneity was high (*p* value for Q test ≤0.05 and *I* ^2^ ≥ 50%), random effects models were used to computed all the results. We conducted subgroup studies (for different study types and different ethnicities) to explore the source of the heterogeneity. In addition, sensitivity analysis was applied to evaluate the stabilization of study. To evaluate publication bias, we conducted Begg's test, Egger's test, and funnel plot. We evaluated methodological quality of studies using JBI criteria.

## 3 RESULTS

### 3.1 Study selection and characteristics

**FIGURE 1:**
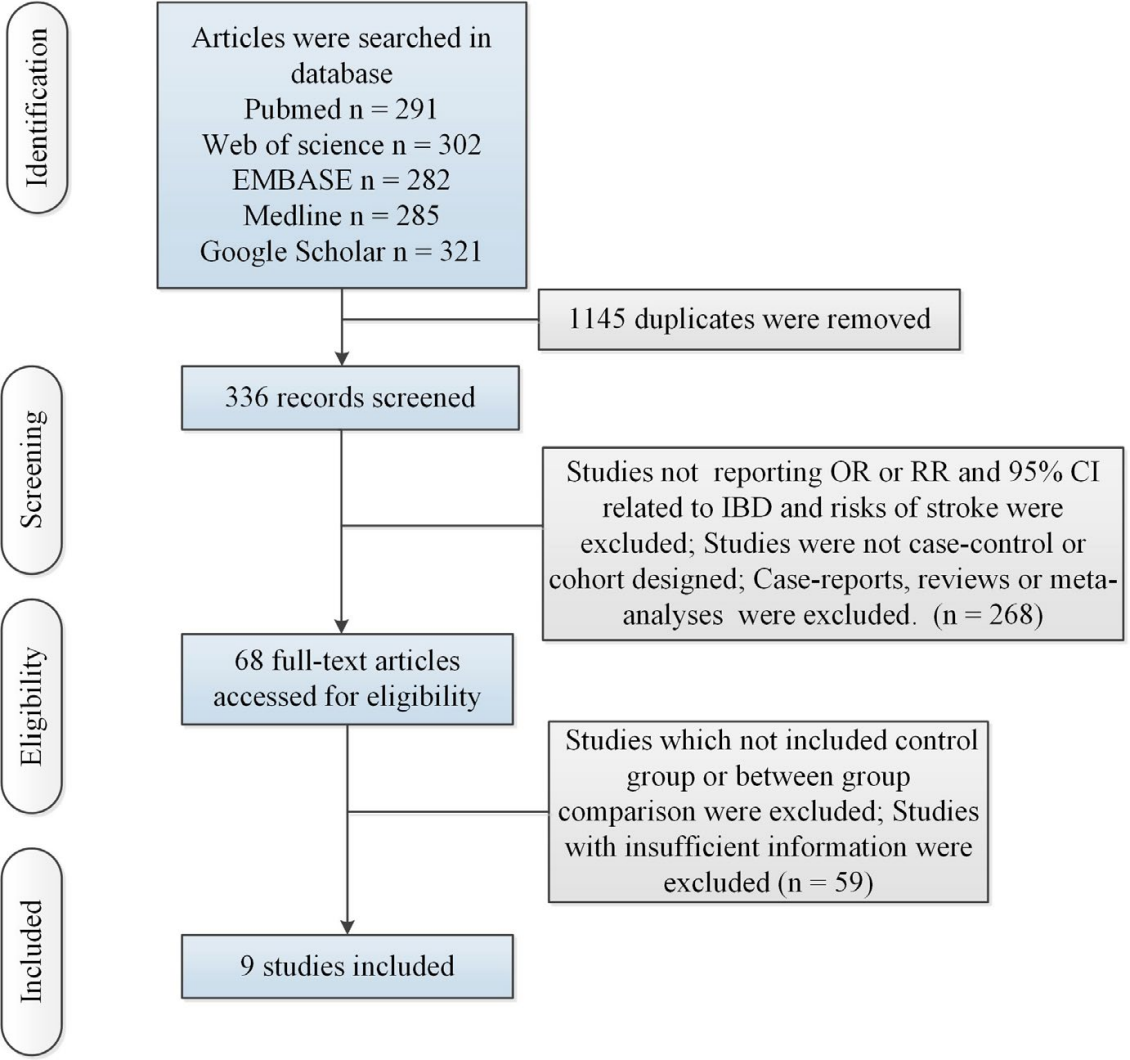
Flow of information through the different phases of a meta‐analysis

Figure 1 showed the selection procedures. Table S1 showed study characteristics. 8 cohort studies (Baena‐Díez et al., 2018; Choi & Lee, 2019; Dregan et al., 2014; Huang et al., 2014; Keller et al., ,^2014^, ^2015^; Kristensen et al., 2013, 2014) (including 641,102 IBD patients) and 1 case–control study (Andersohn et al., 2010) (including 149,908 patients with stroke) were included to explore the association between IBD and risk of stroke.

### 3.2 Results of meta‐analysis

**FIGURE 2:**
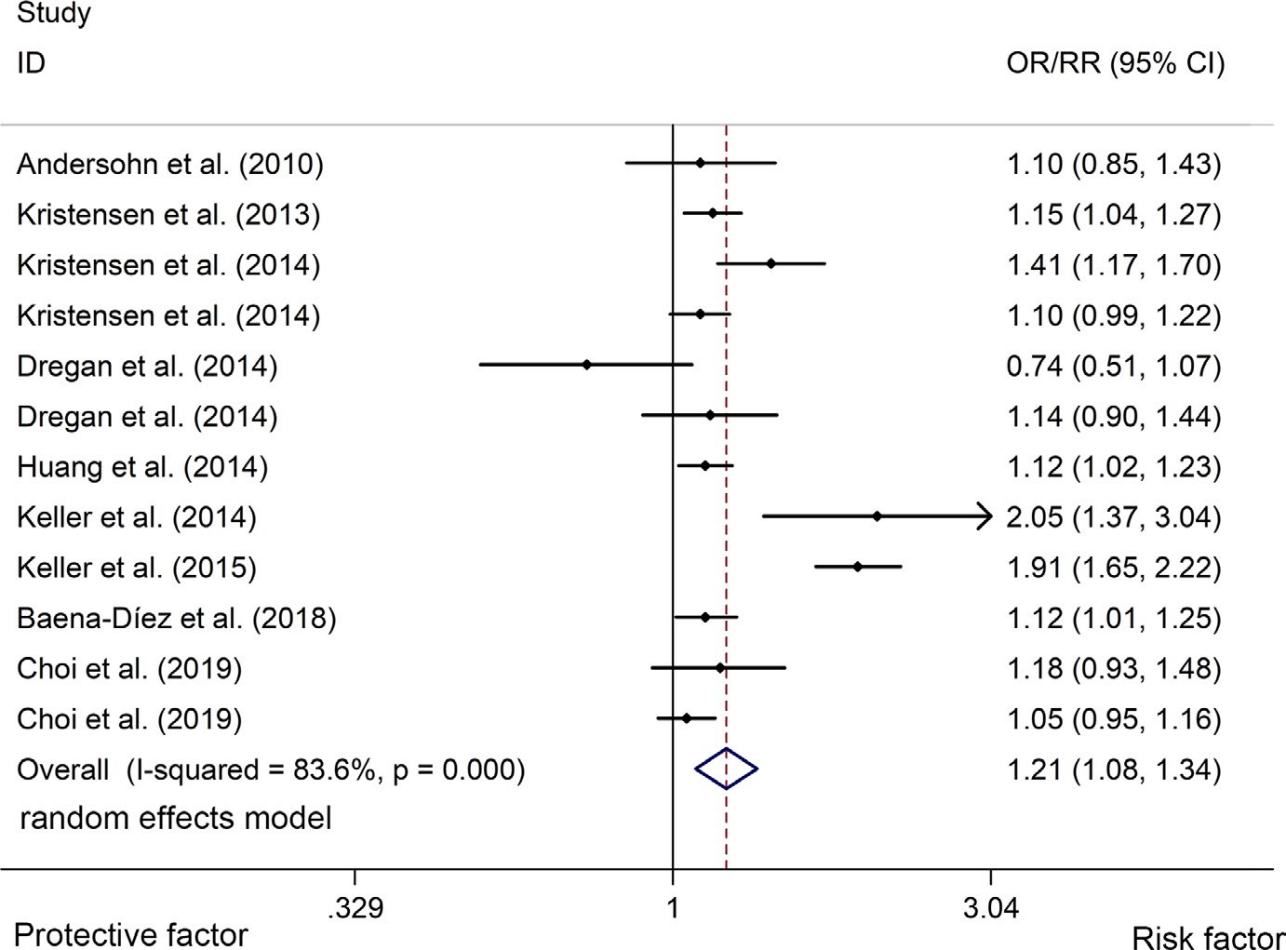
Forest plots of association between IBD and risk of stroke. IBD, inflammatory bowel disease

**FIGURE 3:**
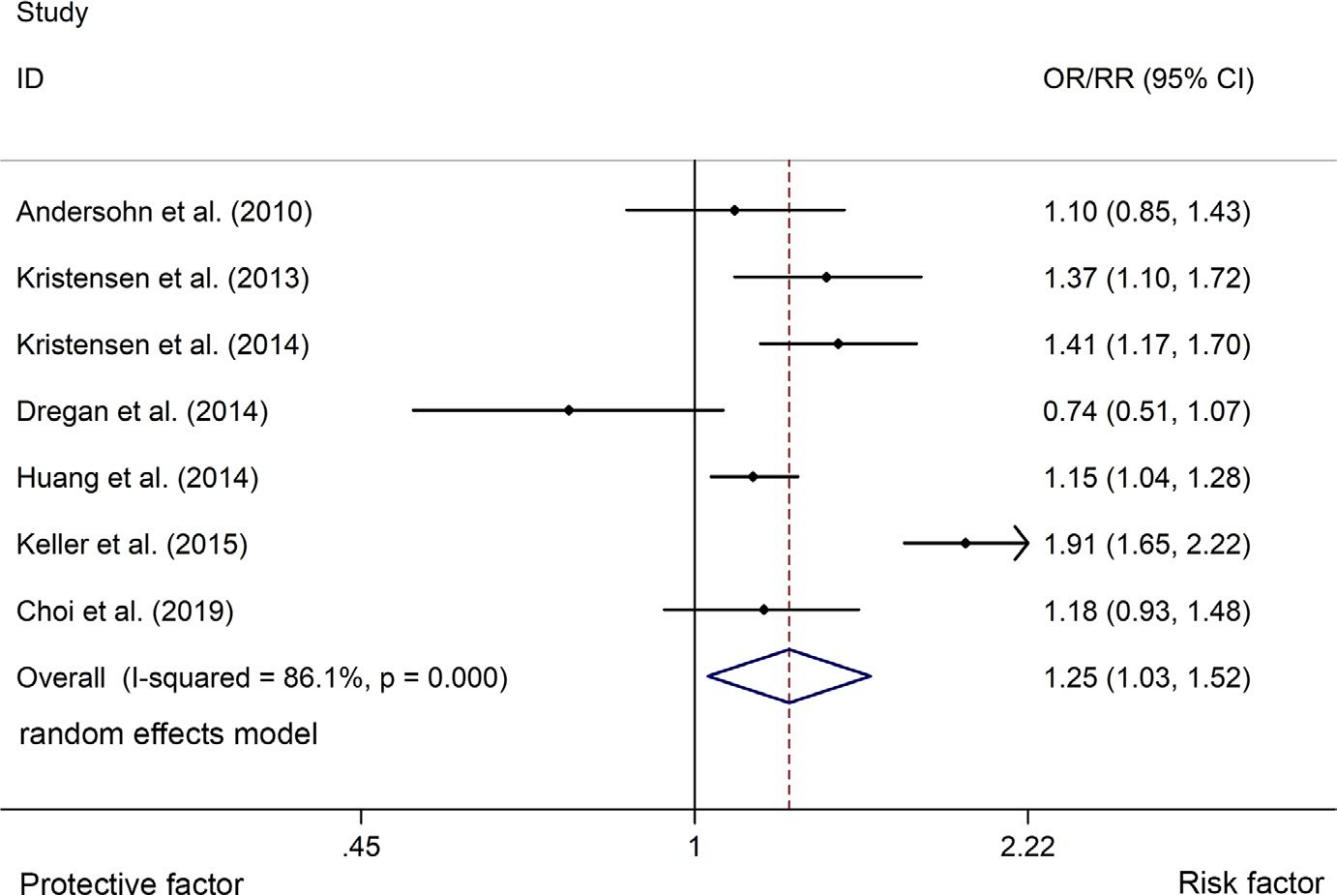
Forest plots of association between CD and risk of stroke. CD, Crohn's disease

**FIGURE 4:**
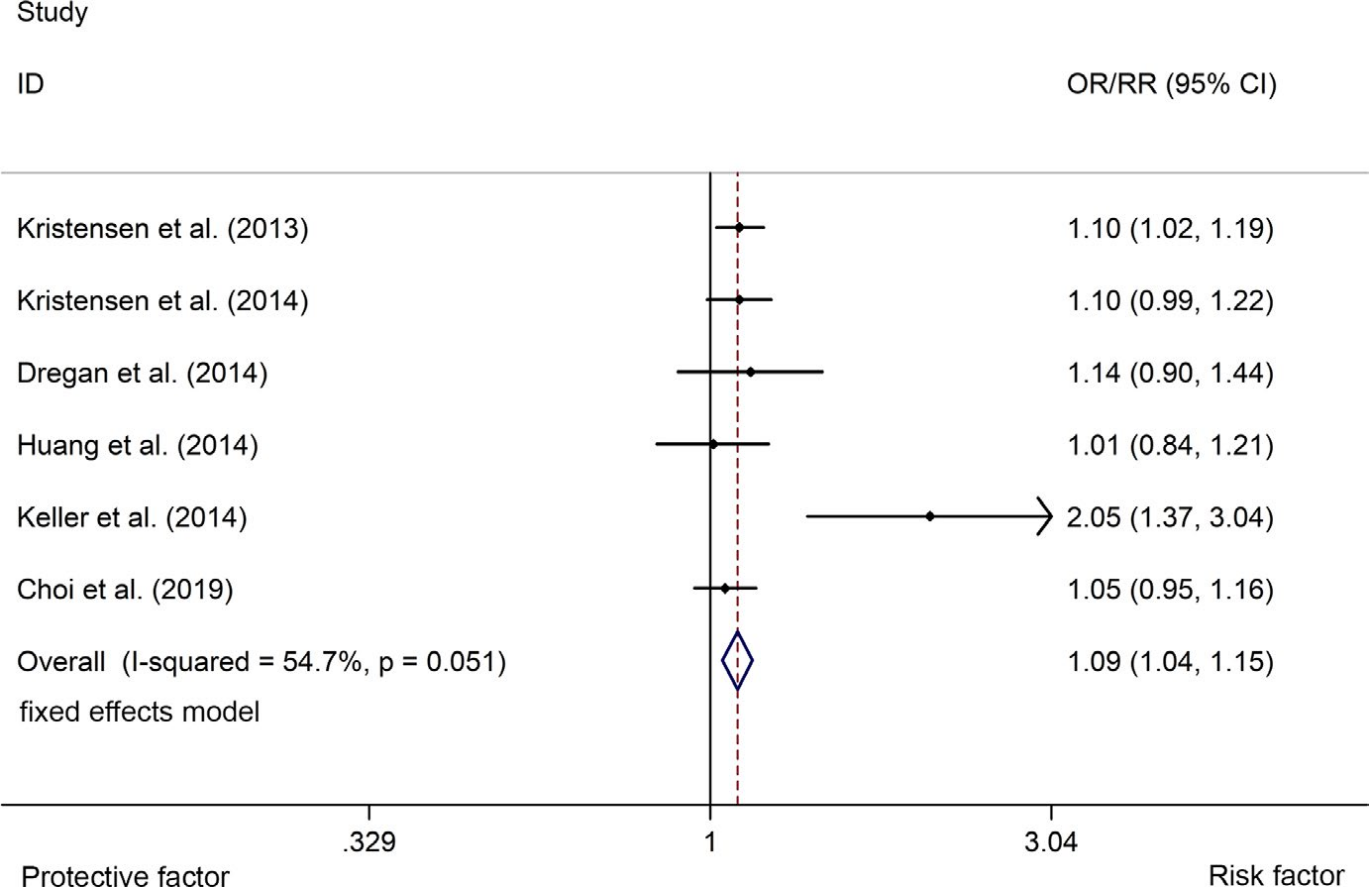
Forest plots of association between UC and risk of stroke. UC, ulcerative colitis

**FIGURE 5:**
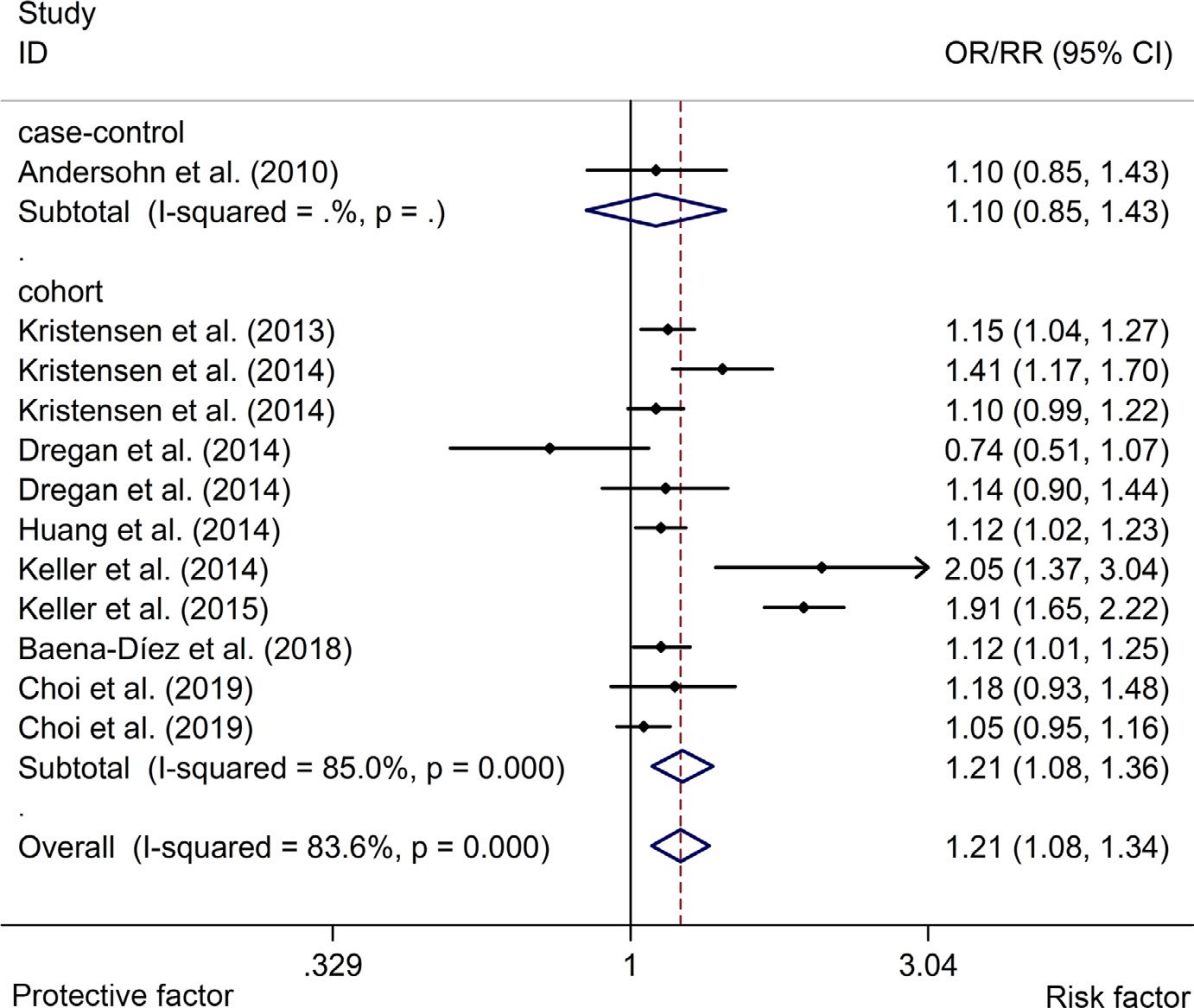
Forest plots of subgroup studies regarding association between IBD and risk of stroke in different ethnicities. IBD, inflammatory bowel disease

**FIGURE 6:**
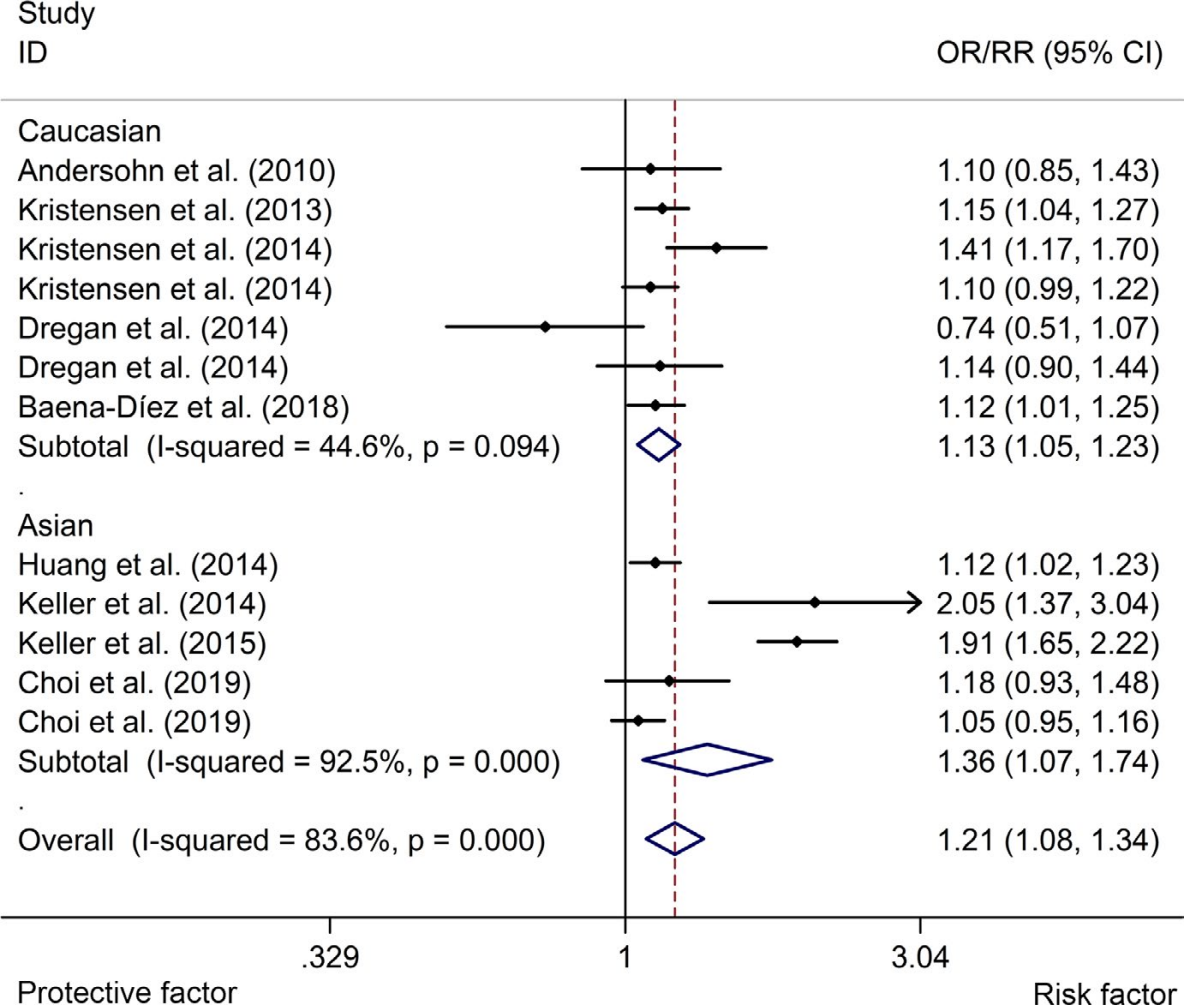
Forest plots of subgroup studies regarding association between IBD and risk of stroke in different study types. IBD, inflammatory bowel disease

**FIGURE 7:**
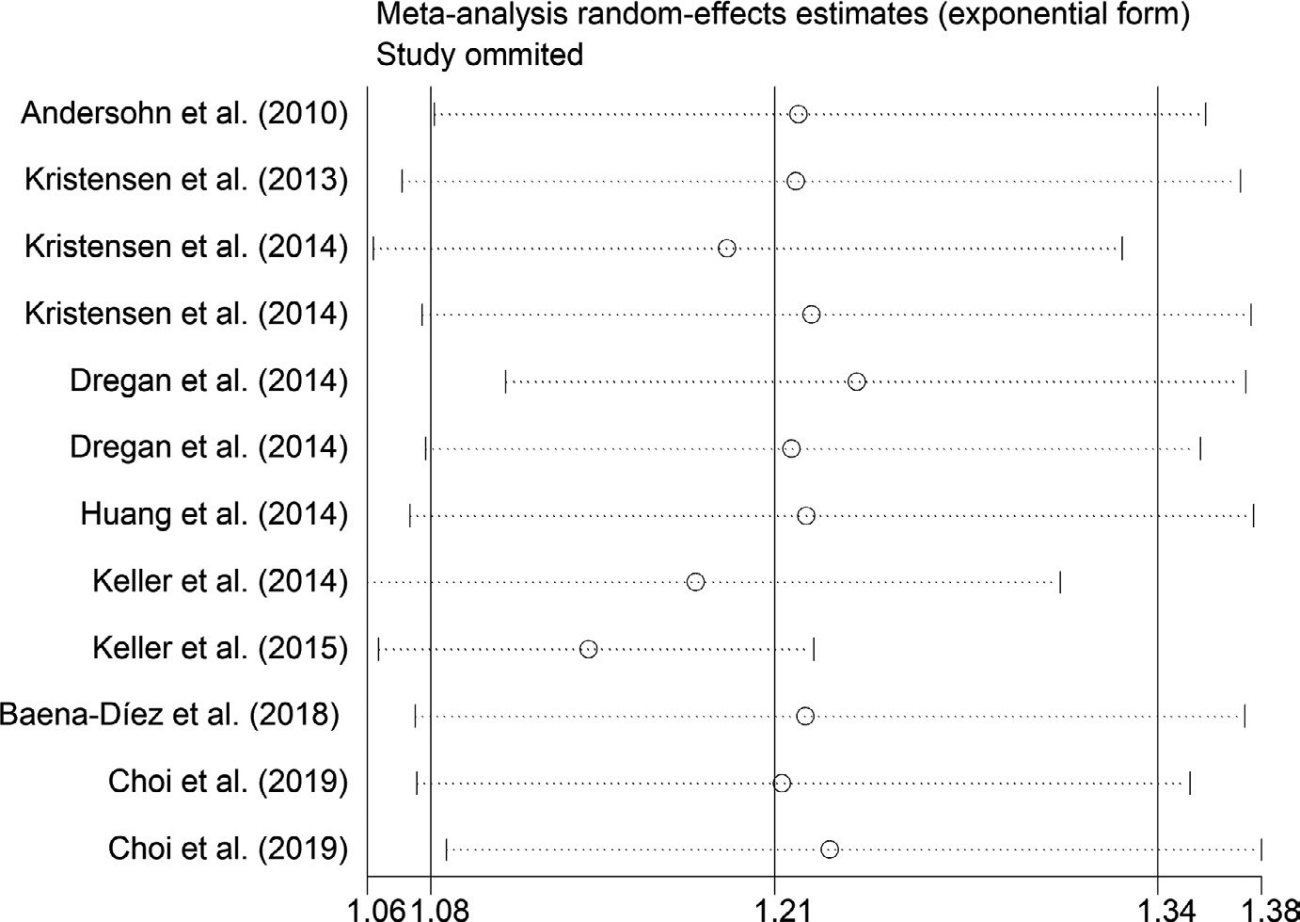
Sensitivity analysis regarding association between IBD and risk of stroke. IBD, inflammatory bowel disease

**FIGURE 8:**
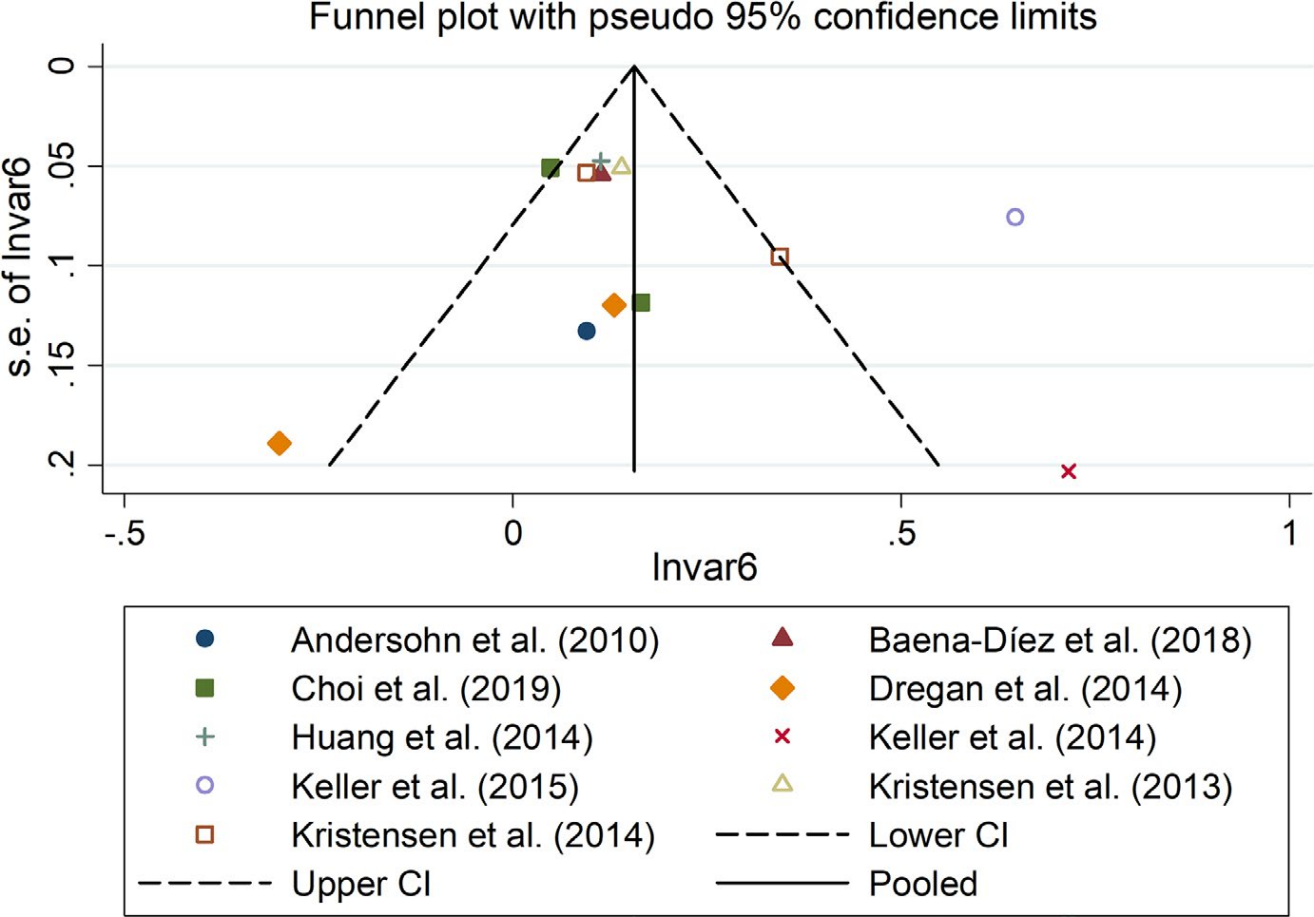
Funnel plots regarding association between IBD and risk of stroke. IBD, inflammatory bowel disease

The meta‐analysis indicated that IBD was associated with an elevated risk of stroke with a random effects model (OR/RR = 1.21, 95% CI 1.08 to 1.34, *I* ^2^ = 83.6%, *p* <.001, Figure 2). In addition, the study showed that CD was associated with a higher risk of stroke with a random effects model (OR/RR = 1.25, 95% CI 1.03 to 1.52, *I* ^2^ = 86.1%, *p* <.001, Figure 3). UC was associated with a higher risk of stroke with a fixed effects model (OR/RR = 1.09, 95% CI 1.04 to 1.15, *I* ^2^ = 54.7%, *p* = .051, Figure 4). Subgroup study showed that IBD was associated with a higher risk of stroke in cohort studies (RR = 1.21, 95% CI 1.08 to 1.36, *I* ^2^ = 85.0%, *p* < .001, Figure 5). Subgroup study showed that IBD was related to an elevated risk of stroke in both Caucasian and Asian groups (Caucasian group: OR/RR = 1.13, 95% CI 1.05 to 1.23, *I* ^2^ = 44.6%, *p* = .094; Asian group: OR/RR = 1.36, 95% CI 1.07 to 1.74, *I^2^* = 92.5%, *p* < .001, Figure 6). In addition, sensitivity analysis indicated no changes in the direction of effect when excluding any one study in all meta‐analyses (Figure 7). Moreover, Begg's test, Egger's test, and funnel plot showed no significant risks of publication bias (Begg's test: *p* = .373; Egger's test: *p* = .433; Figure 8). JBI criteria were attached in Data S1.

## 4 DISCUSSION

Our study included 8 cohort studies and 1 case–control study, with a total of 791,010 samples, and showed that IBD was related to an increased risk of stroke. Our result was consistent with several previous studies (Choi, Lee, et al., 2019; Xiao et al., 2015). The study also showed that CD was more relevant to the risk of stroke than UC. And subgroup study demonstrated that IBD was a risk factor of stroke in both Caucasians and Asians.

Several meta‐analyses also reported the relationship between IBD and other cardiovascular diseases (CVD) such as myocardial infarction and atrial fibrillation (; Panhwar et al., 2019). However, the potential mechanisms of IBD in the development of stroke remain unclear. Published articles indicated that the aberrant immune response to abnormal intestinal microflora in genetically predisposed individuals may lead to the chronic inflammation in IBD (Maloy & Powrie, 2011). The status of low degree chronic inflammation can contribute to the increasing risk of atherosclerosis which is a potential risk factor for the development of CVD (Soysal et al., 2020). The inflammation‐mediated premature atherosclerosis often appears in the patients with IBD, and the biochemical and genetic markers of IBD patients are analogous to the patients with atherosclerotic CVD (Choi, et al., 2019). In addition, chronic inflammation is thought to result in the hypercoagulable state (Levi, 2018). So the role of chronic inflammation of IBD in the progression of IBD‐associated CVD deserves further evaluation. The intestinal microflora are suggested to also potentially contribute to CVD (Choi, et al., 2019). The intestinal microflora and bacterial products may translocate into circulation through impaired intestinal mucosal barrier and intensify inflammatory state (Wu et al., 2017). These factors may serve as the potential mechanism of an increased risk of stroke in IBD patients.

The study included high‐quality published articles about the relationship between IBD and stroke; however, our study has several limitations. First, included studies were from different medical register databases; therefore, diagnostic criteria and disease degree may be different. This might cause clinical heterogeneity. However, the study used random effects models to compute all the results when the heterogeneity was high. In addition, subgroup studies were used to explore the source of the statistical heterogeneity across studies. Second, published studies did not provide patients information in detail such as smoking, alcohol drinking, and use of nonsteroidal anti‐inflammatory drugs. So the studies adjusted for confounding factors for stroke. This restricts a further meta‐regression analysis to explore effect of these indicators on statistical heterogeneity across studies.

In a word, our study showed that IBD is a risk factor for stroke. More high‐quality large‐sample epidemiologic studies about the relationship between IBD and stroke should be further conducted. And the mechanism of IBD in the development of stroke also should be explored in the future.

## CONFLICT OF INTERESTS

No conflict of interests.

### PEER REVIEW

The peer review history for this article is available at https://publons.com/publon/10.1002/brb3.2159.

## Supporting information

Supplementary Material

Supplementary Material
