# Supplementary material for: Increased risk of stroke among patients with inflammatory bowel disease: A PRISMA‐compliant meta‐analysis
Source: Brain Behav. 2021 May 7;11(6):e02159. doi: 10.1002/brb3.2159 (PMC8213927; doi:10.1002/brb3.2159)
Supplement: Supplementary file 2 — Supplementary Material [file BRB3-11-e02159-s001.pdf]

THE JOANNA BRIGGS INSTITUTE

JBI Critical Appraisal Checklist for Systematic Reviews and Research Syntheses

Reviewer Xiang Wang Date 2021-3-28

Author Yao Chen Year 2021 Record Number ______

	Yes	No	Unclear	Not applicable
1. Is the review question clearly and explicitly stated?	✓			
2. Were the inclusion criteria appropriate for the review question?	✓			
3. Was the search strategy appropriate?	✓			
4. Were the sources and resources used to search for studies adequate?	✓			
5. Were the criteria for appraising studies appropriate?	✓			
6. Was critical appraisal conducted by two or more reviewers independently?	✓			
7. Were there methods to minimize errors in data extraction?	✓			
8. Were the methods used to combine studies appropriate?	✓			
9. Was the likelihood of publication bias assessed?	✓			
10. Were recommendations for policy and/or practice supported by the reported data?				✓
11. Were the specific directives for new research appropriate?	✓			

Overall appraisal: Include ✓ Exclude ☐ Seek further info ☐

Comments (Including reason for exclusion)

No comments
